# OsPHR3 affects the traits governing nitrogen homeostasis in rice

**DOI:** 10.1186/s12870-018-1462-7

**Published:** 2018-10-17

**Authors:** Yafei Sun, Wenzhen Luo, Ajay Jain, Lu Liu, Hao Ai, Xiuli Liu, Bing Feng, Liang Zhang, Zhantian Zhang, Xu Guohua, Shubin Sun

**Affiliations:** 1 State Key Laboratory of Crop Genetics and Germplasm Enhancement, Key Laboratory of Plant Nutrition and Fertilization in Low-Middle Reaches of the Yangtze River, Ministry of Agriculture, Nanjing Agricultural University, Nanjing, 210095 China; 2 Institute of Eco-Environment and Plant Protection, Shanghai Academy of Agricultural Sciences, Shanghai, 201403 China; 3 Amity Institute of Biotechnology, Amity University Rajasthan, Kant Kalwar, NH-11C, Jaipur, 303002 India

**Keywords:** Rice, Arabidopsis, Phosphate, Nitrogen variants, *OsPHR3*, Pi availability

## Abstract

**Background:**

Phosphate (Pi) and Nitrogen (N) are essential macronutrients required for plant growth and development. In *Arabidopsis thaliana* (Arabidopsis), the transcription factor *PHR1* acts as a Pi central regulator. *PHL1* is a homolog of *PHR1* and also plays a role in maintaining Pi homeostasis. In rice (*Oryza sativa*), *OsPHR1–4* are the orthologs of *PHR1* and have been implicated in regulating sensing and signaling cascades governing Pi homeostasis.

**Results:**

Here the role of *OsPHR3* was examined in regulating the homeostasis of N under different Pi regimes. Deficiencies of different variants of N exerted attenuating effects on the relative expression levels of *OsPHR3* in a tissue-specific manner*.* For the functional characterization of *OsPHR3*, its Tos17 insertion homozygous mutants i.e., *osphr3–1*, *osphr3–2,* and *osphr3–3* were compared with the wild-type for various morphophysiological and molecular traits during vegetative (hydroponics with different regimes of N variants) and reproductive (pot soil) growth phases. During vegetative growth phase, compared with the wild-type, *OsPHR3* mutants showed significant variations in the adventitious root development, influx rates of ^15^N-NO_3_^−^ and ^15^N-NH_4_^+^, concentrations of total N, NO_3_^−^ and NH_4_^+^ in different tissues, and the relative expression levels of *OsNRT1.1a*, *OsNRT2.4, OsAMT1;1, OsNia1* and *OsNia2.* The effects of the mutation in *OsPHR3* was also explicit on the seed-set and grain yield during growth in a pot soil. Although Pi deficiency affected total N and NO_3_^−^ concentration, the lateral root development and the relative expression levels of some of the NO_3_^−^ and NH_4_^+^ transporter genes, its availability did not exert any notable regulatory influences on the traits governing N homeostasis.

**Conclusions:**

*OsPHR3* plays a pivotal role in regulating the homeostasis of N independent of Pi availability.

**Electronic supplementary material:**

The online version of this article (10.1186/s12870-018-1462-7) contains supplementary material, which is available to authorized users.

## Background

Rice (*Oryza sativa* L.) is the main dietary staple for more than half of the 7.5 billion populations in the world, of which ~ 90% is consumed in Asia alone (www.irri.org/rice-today). United Nations raises world population forecast to 9.8 billion people by 2050 due to escalated population growth particularly in Africa and India (www.un.org). According to FAO, world agriculture will thus face the daunting task of using scarce natural resources more efficiently and adapting to climate change for producing ~ 70% more food for feeding additional 2.3 billion people by 2050 (www.fao.org). Since rice provides 27% and 20% of dietary energy supply and dietary protein intake, respectively in the developing world (www.fao.org), its sustainable production is increasingly becoming pivotal for global food security.

Nitrogen (N) is a key component of important macromolecules such as nucleic acids, proteins and chlorophyll and constitutes ~ 1.5–2% of plant dry matter [1]. N is taken up by plants as nitrate (NO_3_^−^) and ammonium (NH_4_^+^) with the former being the predominant form in most soils [2]. If N deficiency is rampant in rice growing soils, it will affect the growth and development of tillers and panicles and consequently the yield potential. Although N-deficient soils are conventionally enriched with N fertilizers, their excessive usage is uneconomical for sustainable agriculture and also poses a serious threat to the environment [3]. In this context, manipulation of a specific molecular entity through biotechnological intervention is an economically viable and eco-friendly paradigm for engineering rice with higher N use efficiency [4]. Now, a repertoire of genes implicated in regulating acquisition, transportation and utilization of N in rice have been identified [2, 5]. Studies also found that availability of phosphate (Pi), an essential nutrient required for optimal growth and development of plants [6–8] exerted variable influence on the expression of some of the genes involved in the sensing and signaling cascades governing homeostasis of N in rice [9, 10].

Reverse genetics approaches have helped to identify several transcription factors (TFs) that extert regulatory influences on an array of functionally diverse genes involved in the maintenance of N and Pi homeostasis [2, 11]. TFs regulate the expression of the target genes by binding to the *cis*-regulatory specific sequences in their promoters [12]. The TF *PHR1* (*PHOSPHATE STARVATION RESPONSE 1*), a homolog of *PSR1* (*PHOSPHATE STARVATION RESPONSE 1*) in *Chlamydomonas reinhardtii* [13] was positionally cloned and characterized in *Arabidopsis thaliana* [14]. PHR1 has a predicted coiled-coil domain and binds as a dimer to an imperfect palindromic PHR1-specific binding sequence (P1BS; GNATATNC) presenting in the promoters of Pi-starvation induced genes [14]. PHR1 acts as a central regulatory TF, which controls spatiotemporal transcriptional activation and repression of several phosphate-starvation responsive (PSR) genes implicated in signaling and different metabolic pathways during Pi deficiency [15–21]. In addition, PHR1 also interacts with *AtFer1* promoter enriched with P1BS during Pi deficiency [22]. *AtFer1* encodes plastid-located ferritin, a protein nanocage which can store up to 4,500 atoms of Fe^3+^ in its interior that are released in a controlled fashion [23]. In Arabidopsis, PHR1 also plays a pivotal role in regulating sulfate flux from shoot to root during Pi deprivation [24] and exerts influence on the crosstalk between Pi and Zn [25]. These studies thus highlighted a key role of PHR1 in regulating the homeostasis of Pi and other essential nutrients. Further, a search for T-DNA mutations at *PHR1*-related genes in public databases led to the identification of *PHR1-LIKE1* (*PHL1*, At5g29000). Pi accumulation was significantly higher in *PHR1*-overexpressing transgenic lines compared with *phl1* mutant, and it was significantly lower in the double mutant *phr1phl1* compared with the latter, which suggested partial functional redundancy between *PHR1* and *PHL1* [16]. In Arabidopsis, *PHL2* and *PHL3* are the homologs of *PHL1* and of these *PHL2* play a pivotal role in regulating transcriptional response to Pi deficiency and is functionally redundant with *PHR1* [21]. In rice, phylogenetic and mutational analyses revealed functional redundancy across *PHR1* orthologs (*OsPHR1–3*) and together they formed a network for regulating sensing and signaling cascades governing Pi homeostasis [26, 27]. Pi-starvation induced *OsPHR4* mediates Pi homeostasis and plays a pivotal role in the regulation of downstream PSR genes [28]. Although the expression of *OsPHR3* is induced by Pi starvation, its mutation does not exert any significant influence on the Pi concentration and on the expression of downstream PSR genes [27]. The study also revealed that OsPHR3 exhibited lowest binding affinity towards P1BS but still plays a role in growth of Pi-deprived Arabidopsis. However, it is not known whether *OsPHR3* plays a role in exerting a regulatory influence on the morphophysiological and molecular traits governing N homeostasis in a manner dependent or independent of Pi availability.

Here, in our study, we showed that *OsPHR3* is responsive to different forms of N irrespective of Pi regimes*.* The silencing of *OsPHR3* triggered wide-spectrum effects on different traits during vegetative and reproductive growth phases. Availability of Pi did not exert any notable effects on *OsPHR3*-mediated regulatory influence on N homeostasis under different N variants and the lateral root development responses under different NO_3_^−^ treatments.

## Results

### *OsPHR3* is responsive to different forms of N

**Fig. 1:**
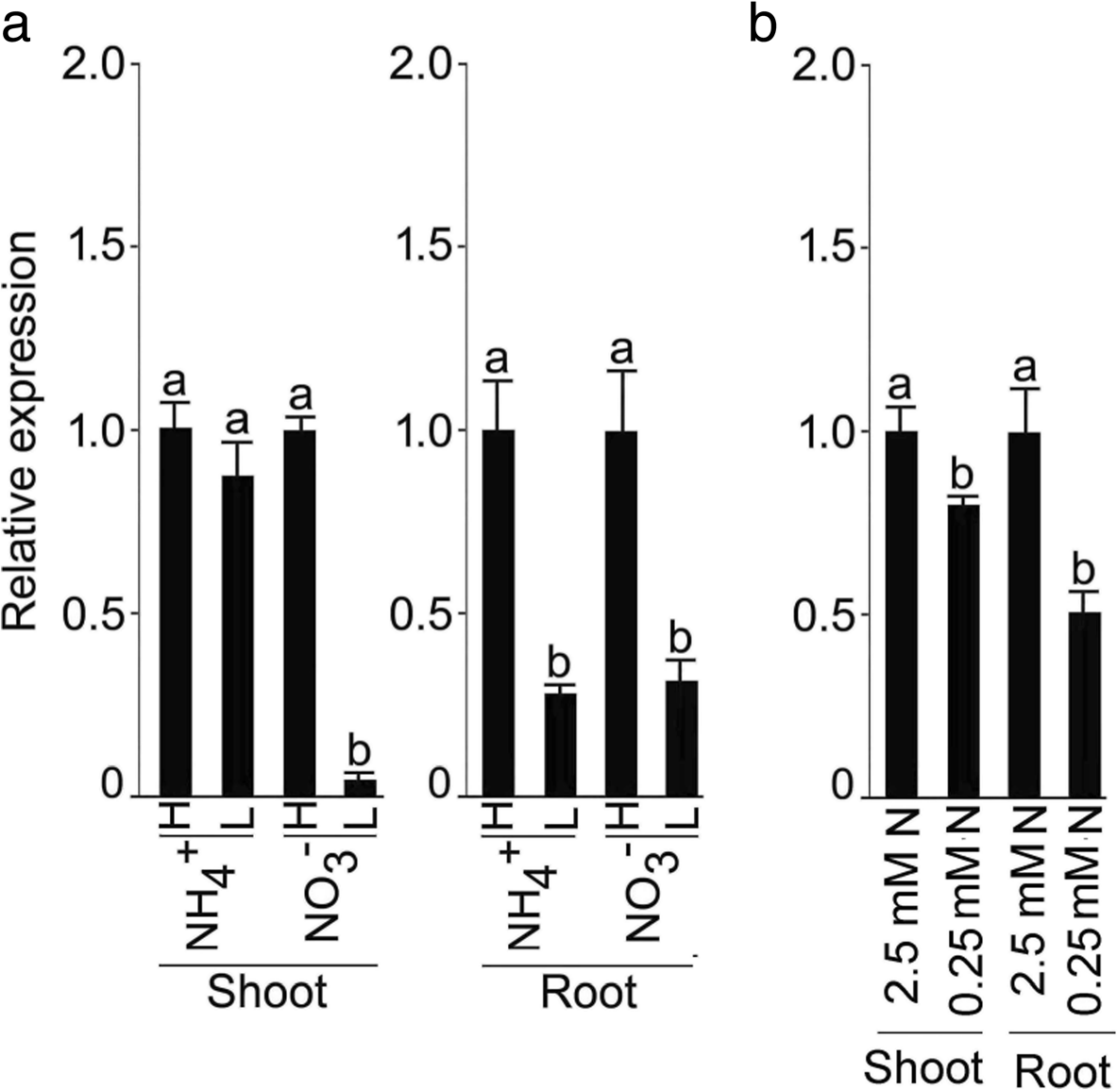
Tissue-specific differential relative expression levels of *OsPHR3* during growth under different regimes of N variants. Seeds of the wild-type were grown hydroponically in IRRI solution for 2 weeks, starved for N for 3 d and then supplied for 24 h with nutrient solution containing high NH_4_^+^ (H NH_4_^+^, 5 mM), low NH_4_^+^ (L NH_4_^+^, 0.25 mM), high NO_3_^−^ (H NO_3_^−^, 5 mM), low NO_3_^−^ (L NO_3_^−^, 0.25 mM), 2.5 mM N (1.25 mM NH_4_^+^ and 1.25 mM NO_3_^−^,) and 0.25 mM (0.125 mM NH_4_^+^ and 0.125 mM NO_3_^−^). Root and shoot were harvested for the qRT-PCR analysis of the relative expression levels of *OsPHR3* in (**a**) high and low NH_4_^+^ or NO_3_^−^ and (**b**) 2.5 mM N and 0.25 mM N conditions. *Actin* (*OsRac1*; LOC_Os03g50885) was used as an internal control and the values for H NH_4_^+^, H NO_3_^−^ and + N were normalized to 1. Values are means ±SE (*n* = 3) and different letters on the histograms indicate that the values differ significantly (*P* < 0.05, one-way ANOVA)

TBLASTN (http://www.ncbi.nlm.nih.gov/BLAST) was employed for searching the homolog of Arabidopsis *AtPHL1* (At5g29000) in rice, which resulted in the identification of *OsPHL1* on the chromosome 2. However, this gene has been reported in 2015, which named as *OsPHR3* (LOC_Os02g04640) [27]. Thus we changed *OsPHL1* to *OsPHR3*. *OsPHR3* is a MYB coiled-coil (MYB-CC) domain-containing TF (http://www.ebi.ac.uk/interpro/). *OsPHR3's*  orthologs are *AtPHR1* and *AtPHL1–3* in Arabidopsis [14, 16, 21] and paralogs are *OsPHR1–4* in rice [26–28]. The amino acid sequence identity of OsPHR3 ranged from 56.96% with OsPHR4 to 26.06% with OsPHR2 (Additional file 1). Multiple amino acid sequence alignment of OsPHR3 with other MYB-CC family members (AtPHR1, AtPHL1, OsPHR1, 2 and 4) revealed the conserved MYB helix-turn-helix (MYB-HTH) and MYB-CC domains (Additional file 1). The qRT-PCR was employed to determine the relative expression levels of *OsPHR3* in the shoot and root of the wild-type rice seedlings grown hydroponically in a medium supplemented with different forms and concentrations of N (H NH_4_^+^/L NH_4_^+^, H NO_3_^−^/L NO_3_^−^ and + N/-N, +N and -N indicate 2.5 mM and 0.25 mM N, respectively) (Fig. 1). The relative expression levels of *OsPHR3* were significantly reduced in the root under L NH_4_^+^, and both shoot and root under L NO_3_^−^ compared with their corresponding H NH_4_^+^ and H NO_3_^−^ (Fig. 1a). Further, the relative expression levels of *OsPHR*3 were significantly attenuated in -N shoot and root compared with +N seedling (Fig. 1b). It was evident from the results that different forms and regimes of N exerted significant influence on the relative expression levels of *OsPHR3* in a tissue-specific manner.

### Silencing of *OsPHR3* affects vegetative growth under different regimes and forms of N and reproductive growth at grain-filling stage

**Fig. 2:**
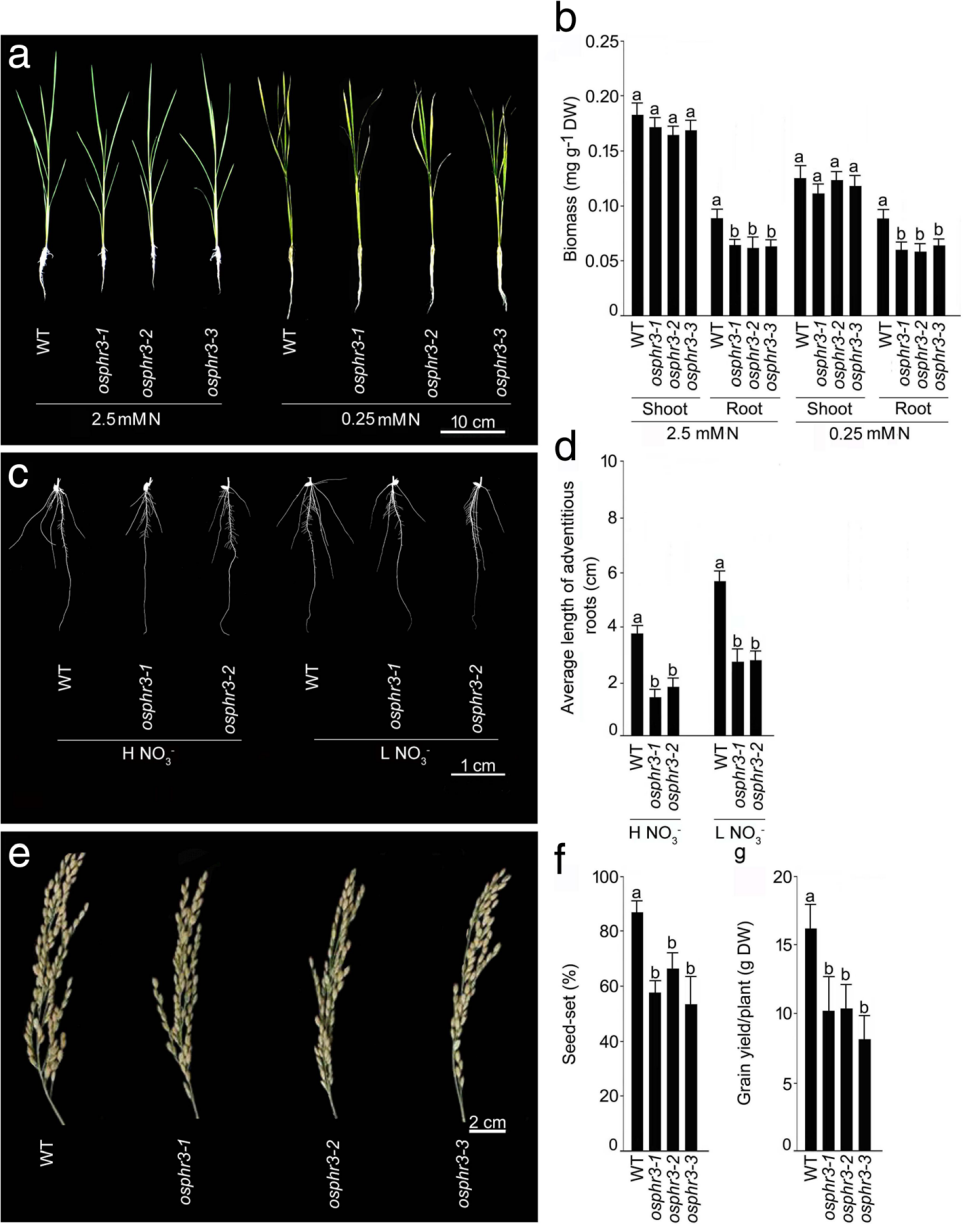
Mutation in *OsPHR3* affects vegetative and reproductive traits under different N and NO_3_^−^ regimes. Seeds of the WT and *OsPHR3* mutants (*osphr3–1, osphr3–2* and *osphr3–3*) were grown hydroponically in IRRI solution for 2 weeks. Seedlings were then transferred to (**a**, **b**) 2.5 mM N and 0.25 mM N and (**c**, **d**) H NO_3_ and L NO_3_^−^ media for 7 d. **e**, **f** WT and the mutants were also grown in a pot soil for 17 weeks (grain-filling stage). Phenotypes of the (**a**) seedlings, (**c**) root system architecture and (**e**) panicles were observed. Data are presented for (**b)** biomass, (**d**) average length of adventitious roots, (**f** )per cent seed-set and (**g**) grain yield/plant. Values in (**b, d** and **e**) are means ±SE (*n* = 5) and different letters on the histograms indicate that the values differ significantly (*P* < 0.05, one-way ANOVA)

Three homozygous *OsPHR3* mutants in the Nipponbare background (*osphr3–1*, *osphr3–2* and *osphr3–3*) were obtained from the rice *Tos*17 insertion mutant database (https://tos.nias.affrc.go.jp) (Additional file 2). There was a *Tos17* insertion in the first (*osphr3–2* and *osphr3–3*) and the last (*osphr3–1*) exon of *OsPHR3* (Additional file 2). Semi-quantitative RT-PCR analysis revealed the absence of *OsPHR3* transcript in these mutants (Additional file 2). These knock-out mutants were then compared with the wild-type for the effects of different N forms and regimes on the vegetative traits (biomass and an average length of the adventitious roots) when grown hydroponically, and also on the reproductive traits (per cent seed-set and grain yield/plant) during growth in a pot soil up to grain-filling stage (Fig. 2). There were no apparent effects on the growth response of the mutant (*osphr3–1*, *osphr3–2* and *osphr3–3*) seedlings compared with the wild-type under both +N and -N conditions (+N and -N indicate 2.5 mM and 0.25 mM N, respectively) (Fig. 2a). Although shoot biomass of the mutants (*osphr3–1*, *osphr3–2* and *osphr3–3*) was comparable with the wild-type irrespective of N regimes, their root biomass was significantly lower than the wild-type under both +N (~ 27–30%) and -N (~ 27–33%) conditions (Fig. 2b). Root system architecture and the primary root length of the mutants (*osphr3–1*and *osphr3–2*) were comparable with the wild-type under both H NO_3_^−^ and L NO_3_^−^ conditions (Fig. 2c). However, an average length of the adventitious roots of the mutants (*osphr3–1*and *osphr3–2*) revealed significant reductions under H NO_3_^−^ (~ 52–62%), L NO_3_^−^ (~ 51–52%) (Fig. 2d) and L NH_4_^+^ (~ 23%) (Additional file 3) conditions compared with the wild-type. To further determine the role of *OsPHR3,* if any, during the reproductive growth phase, the wild-type and the mutants (*osphr3–1*, *osphr3–2* and *osphr3–3*) were grown in a pot soil up to the grain-filling stage (Fig. 2e-g). The growth of the panicle was retarded in the mutants compared with the wild-type (Fig. 2e), which was congruent with significant reductions in the per cent seed-set (~ 23–35%) (Fig. 2f), and grain yield/plant (~ 35–49%) (Fig. 2g). The results suggested a broad spectrum positive regulatory influence of *OsPHR3* during both vegetative and reproductive growth phases of rice.

### Silencing of *OsPHR3* affects N homeostasis

**Fig. 3:**
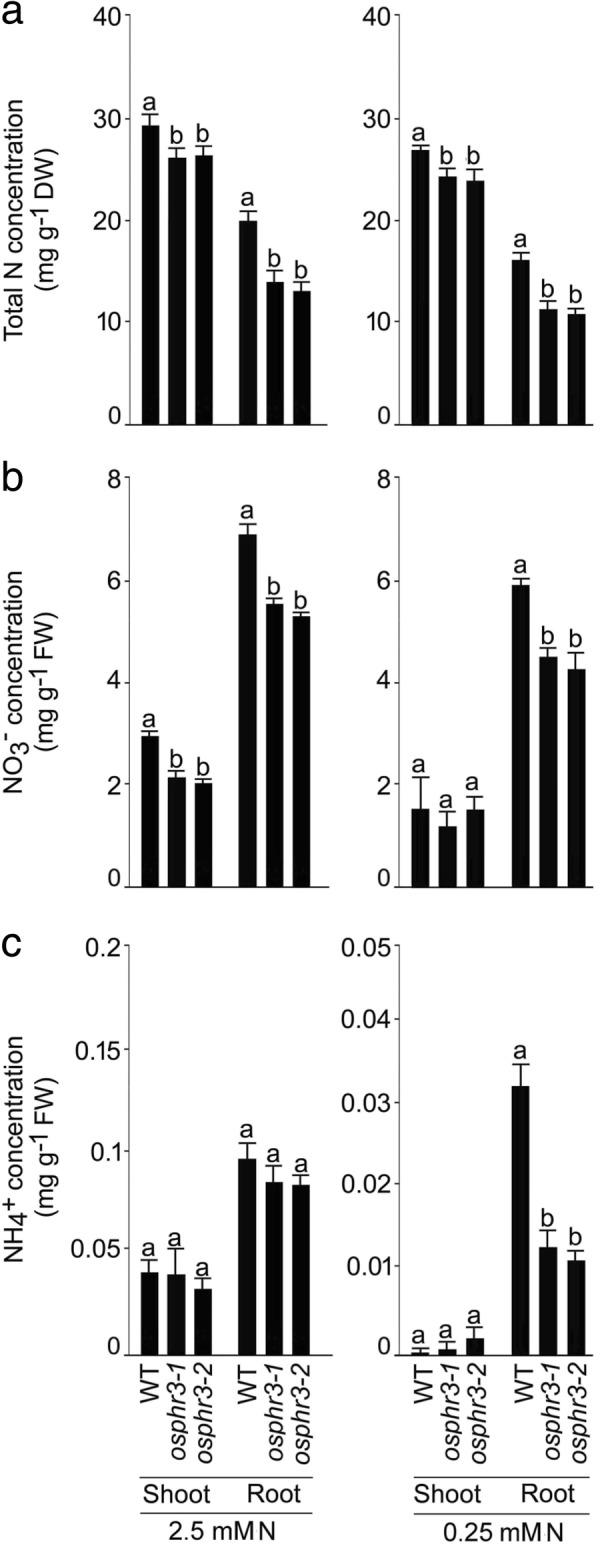
Mutation in *OsPHR3* affects total N, NO_3_^−^ and NH_4_^+^ concentrations under different N regimes. Seeds of the WT and mutants (*osphr3–1* and *3–2*) were grown hydroponically in IRRI solution for 2 weeks, deprived of N for 3 d and then transferred to 2.5 mM N and 0.25 mM N media for 7 d. Shoot and root were harvested. Data are presented for the concentration of (**a)** total N, (**b**) NO_3_^−^ and (**c**) NH_4_^+^. Values are means ±SE (*n* = 5) and different letters on the histograms indicate that the values differ significantly (*P* < 0.05, one-way ANOVA)

**Fig. 4:**
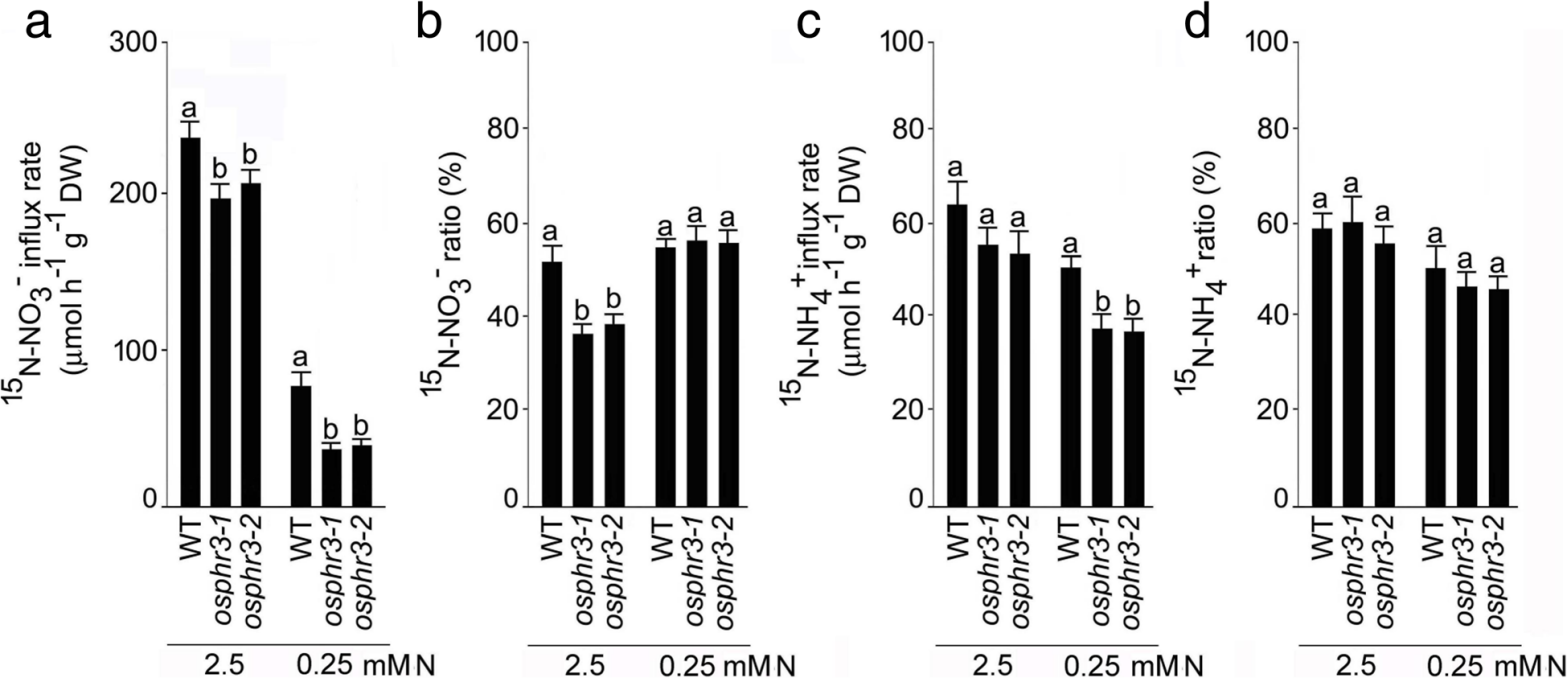
Mutation in *OsPHR3* affects influx and translocation of ^15^N-NO_3_^−^ and ^15^N-NH_4_^+^ under different N regimes. Seeds of the WT and mutants (*osphr3–1* and *3–2*) were grown hydroponically in IRRI solution for 2 weeks and then deprived of N for 3 d. Seedlings were then treated with ^15^N-NO_3_^−^ and ^15^N-NH_4_^+^ under 2.5 mM N and 0.25 mM N conditions for 10 min and 1 d for determining their influx and translocation, respectively. The influx rate of (**a**) ^15^N-NO_3_^−^ and (**c**) ^15^N-NH_4_^+^. The translocation ratio of (**b**) ^15^N-NO_3_^−^ and (**d**) ^15^N-NH_4_^+^. Values are means ±SE (*n* = 5) and different letters on the histograms indicate that the values differ significantly (*P* < 0.05, one-way ANOVA)

**Fig. 5:**
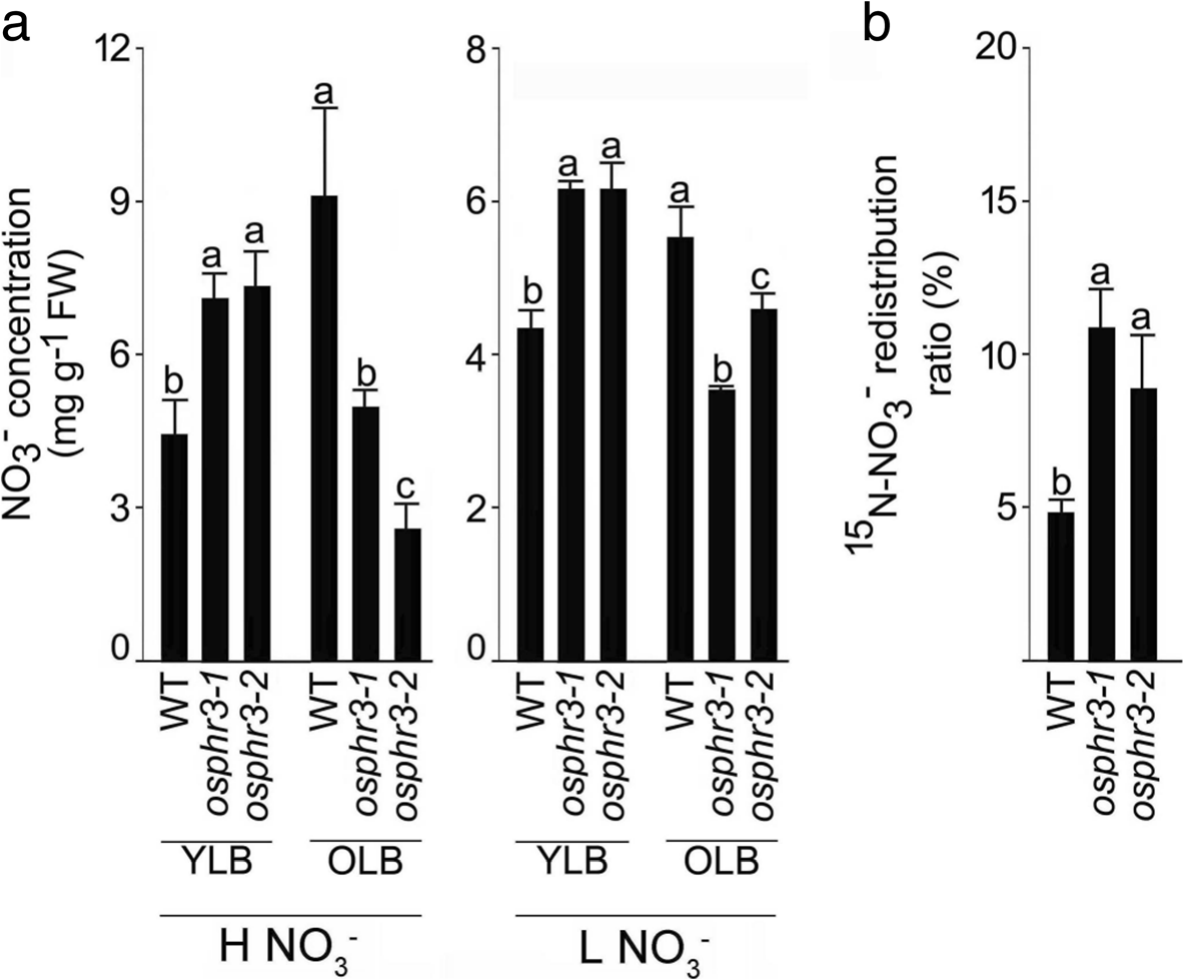
Mutation in *OsPHR3* affects concentration and redistribution of NO_3_^−^ in young and old leaf blades. Seeds of the WT and mutants (*osphr3–1* and *3–2*) were grown hydroponically in IRRI solution for 3 weeks and then deprived of N for 3 d. **a** Seedlings were then transferred to H NO_3_^−^ and L NO_3_^−^ for 7 d. Young leaf blade (YLB) and old leaf blade (OLB) were harvested and assayed for NO_3_^−^ concentration. **b** For determining ^15^NO_3_^−^ redistribution ratio, the oldest leaf blade of the WT and mutant seedlings were incubated in a solution containing ^15^N-NO_3_^−^ (5 mM) for 5 h. values are means ±SE (*n* = 5) and different letters on the histograms indicate that the values differ significantly (*P* < 0.05, one-way ANOVA). FW, fresh weight

The wild-type and the mutants (*osphr3–1* and *3–2*) were grown hydroponically under +N and -N condition for 7 d to determine the effects of the mutation in *OsPHR3* on the concentrations of total N, NO_3_^−^ and NH_4_^+^ in the shoot and root of the seedlings (+N and -N indicate 2.5 mM and 0.25 mM N, respectively) (Fig. 3). There were no significant differences in the concentrations of NO_3_^−^ in -N shoot, NH_4_^+^ in +N and -N shoot and + N root of the wild-type and the mutants (Fig. 3a-c). However, the attenuating effects of the mutation in *OsPHR3* were evident on the concentrations of total N in +N shoot (~ 9–11%) and -N shoot (~ 11–13%), +N root (~ 30–38%) and -N root (~ 27–30%), NO_3_^−^ in +N shoot (~ 26–30%), +N root (~ 19–23%) and -N root (~ 23–37%) and NH_4_^+^ in -N root (~ 60–64%) (Fig. 3a-c). Further, the wild-type and the mutants (*osphr3–1* and *3–2*) were grown in a pot soil up to the maturity (grain harvest stage) to determine the effects of the mutation in *OsPHR3* on the concentration of total N in different tissues at the reproductive stage (Additional file 4). Total N concentration was comparable in the 3rd leaf blade, culm, leaf sheath, panicle, significantly lower in the 6th leaf blade (~ 17–22%), and significantly higher in the 1st leaf blade (~ 17–21%) and seed (~ 12–16%). Isotope assays were then employed for comparing the influx of NO_3_^−^ and NH_4_^+^ for 10 min and their subsequent translocation to the shoot after 24 h between the wild-type and the mutants (*osphr3–1* and *3–2*) grown hydroponically under different N regimes (Fig. 4). Compared with the wild-type, the mutants showed significantly lower influx rate of ^15^NO_3_^−^ (~ 12–17%) in +N root, ^15^NO_3_^−^ (~ 49–50%) and ^15^NH_4_^+^ (~ 25–27%) in -N root, while the corresponding values remained comparable of ^15^NH_4_^+^ in the +N root (Fig. 4a, c). Although the ratio (translocation) of ^15^NO_3_^−^ in -N plant and ^15^NH_4_^+^ in +N and -N plant in the wild-type and the mutants were comparable, the ratio of ^15^NO_3_^−^ in +N plant was significantly lower (~ 25–29%) in the mutants compared with the wild-type (Fig. 4b, d). In addition, the concentration of NO_3_^−^ was assayed in the second young leaf blade (YLB) and fourth old leaf blade (OLB) at a five-leaf stage of the wild-type and mutants (*osphr3–1* and *osphr3–2*) grown hydroponically under H NO_3_^−^ and L NO_3_^−^ condition for 7d (Fig. 5a). Compared with the wild-type, NO_3_^−^ concentrations in the mutants were ~ 59–85% and ~ 37% higher in YLB under H NO_3_^−^ and L NO_3_^−^, respectively. On the contrary, an opposite trend was observed in OLB, where the values were ~ 45–71% and ~ 16–33% lower under H NO_3_^−^ and L NO_3_^−^, respectively. Finally, redistribution of NO_3_^−^ from the older to younger leaf was assayed in the wild-type and the mutants (*osphr3–1* and *osphr3–2*) by exposing the N-starved oldest leaves to ^15^N-NO_3_^−^ for 5 h (Fig. 5b). The mutants showed significantly higher (~ 84–125%) redistribution ratio of ^15^NO_3_^−^ compared with the wild-type. The results thus suggested the regulatory influence of *OsPHR3* in the maintenance of homeostasis of diverse forms of N under different N regimes in a tissue-specific manner.

### Silencing of *OsPHR3* differentially affects the expression of NO_3_^−^ and NH_4_^+^ transporter and NO_3_^−^ reductase genes under different N regimes

**Fig. 6:**
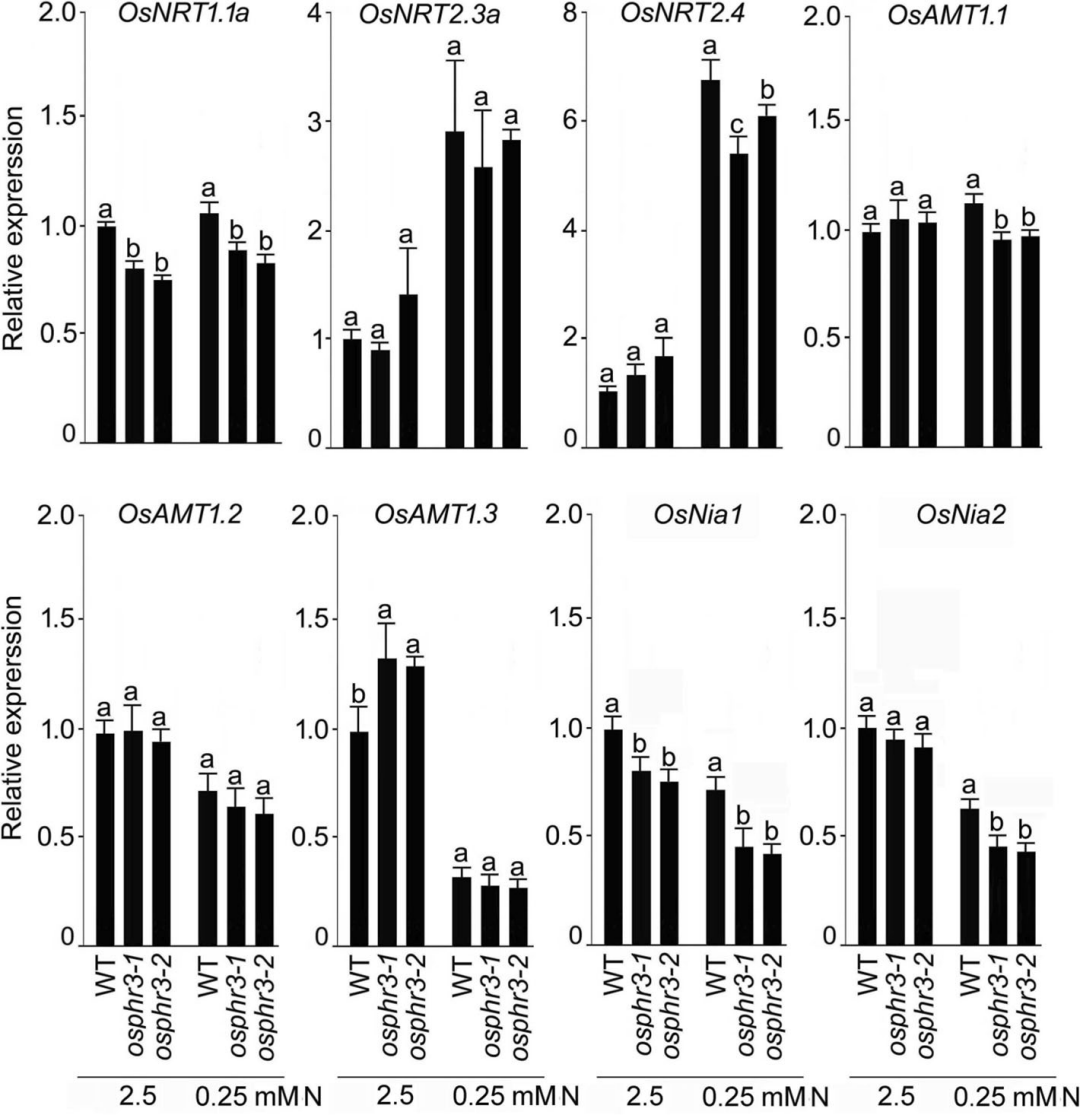
Mutation in *OsPHR3* affects the expression of NO_3_^−^ and NH_4_^+^ transporter and nitrate reductase genes. Seeds of the WT and mutants (*osphr3–1* and *3–2*) were grown hydroponically in IRRI solution for 2 weeks, deprived of N for 3 d and then transferred to 2.5 mM N and 0.25 mM N media for 1 d. Roots were harvested and qRT-PCR was employed for determining the relative expression levels of genes encoding NO_3_^−^ (*OsNRT1.1a*, *OsNRT2.3a* and *OsNRT2.4*), NH_4_^+^ (*OsAMT1.1*, *OsAMT1.2* and *OsAMT1.3*) transporters and nitrate reductase (*OsNia1* and *OsNia2*). *Actin* was used as an internal control and + N WT values were normalized to 1. Values are means ±SE (*n* = 3) and different letters on the histograms indicate that the values differ significantly (*P* < 0.05, one-way ANOVA)

Since the mutation in *OsPHR3* exerted significant influences on the concentrations of total N, NO_3_^−^ and NH_4_^+^ (Fig. 3), the influx rate and translocation of NO_3_^−^ and NH_4_^+^ (Fig. 4) and the concentration and remobilization of NO_3_^−^ from OLB to YLB (Fig. 5), it raised a pertinent question about its likely influence on the relative expression of the genes implicated in sensing and signaling cascades governing N homeostasis. Several genes have been identified that play pivotal roles in N assimilation and use efficiency [2]. Among these genes, those encoding for transporters for NO_3_^−^ (*OsNRTs*) [29–33] and NH_4_^+^ (*OsAMTs*) [34–36] have been functionally characterized. NO_3_^−^ reductase genes (*OsNia1* and *OsNia2*) play a role in converting NO_3_^−^ to NH_4_^+^ in roots, which related to N metabolism [2]. The expression pattern of the NO_3_^−^ reductase genes are well known to be low during nitrate deficiency and high in nitrate-sufficiency [37]. Therefore, qRT-PCR was employed to determine the effects of the mutation in *OsPHR3* on the relative expression levels of NO_3_^−^ (*OsNRT1.1a*, *OsNRT2.3a* and *OsNRT2.4*) and NH_4_^+^ (*OsAMT1.1*, *OsAMT1.2*, and *OsAMT1.3*) transporter and NO_3_^−^ reductase (*OsNia1* and *OsNia2*) genes in the roots of the wild-type and the mutants (*osphr3–1* and *osphr3–2*) grown hydroponically under +N and -N conditions (Fig. 6). The relative expression levels of these genes were comparable in the wild-type and the mutants under +N (*OsNRT2.4* and *OsAMT1.1*), -N (*OsAMT1.3*) or both under +N and -N conditions (*OsNRT2.3a* and *OsAMT1.2*) (+N and -N indicate 2.5 mM and 0.25 mM N, respectively). On the contrary, the relative expression levels of *OsNRT1.1a* and *OsNia1* under +N and -N condition, and those of *OsNRT2.4, OsAMT1.1* and *OsNia2* under -N condition were significantly attenuated in the mutants compared with the wild-type. The relative expression level of *OsAMT1.3* under +N condition was significantly augmented compared with the wild-type. The results suggested differential regulatory influences of *OsPHR3* on the relative expression levels of NO_3_^−^ and NH_4_^+^ transporter and NO_3_^−^ reductase genes under different N regimes.

### *OsPHR3* affects lateral root development under different NO_3_^−^ regimes independent of pi availability

**Fig. 7:**
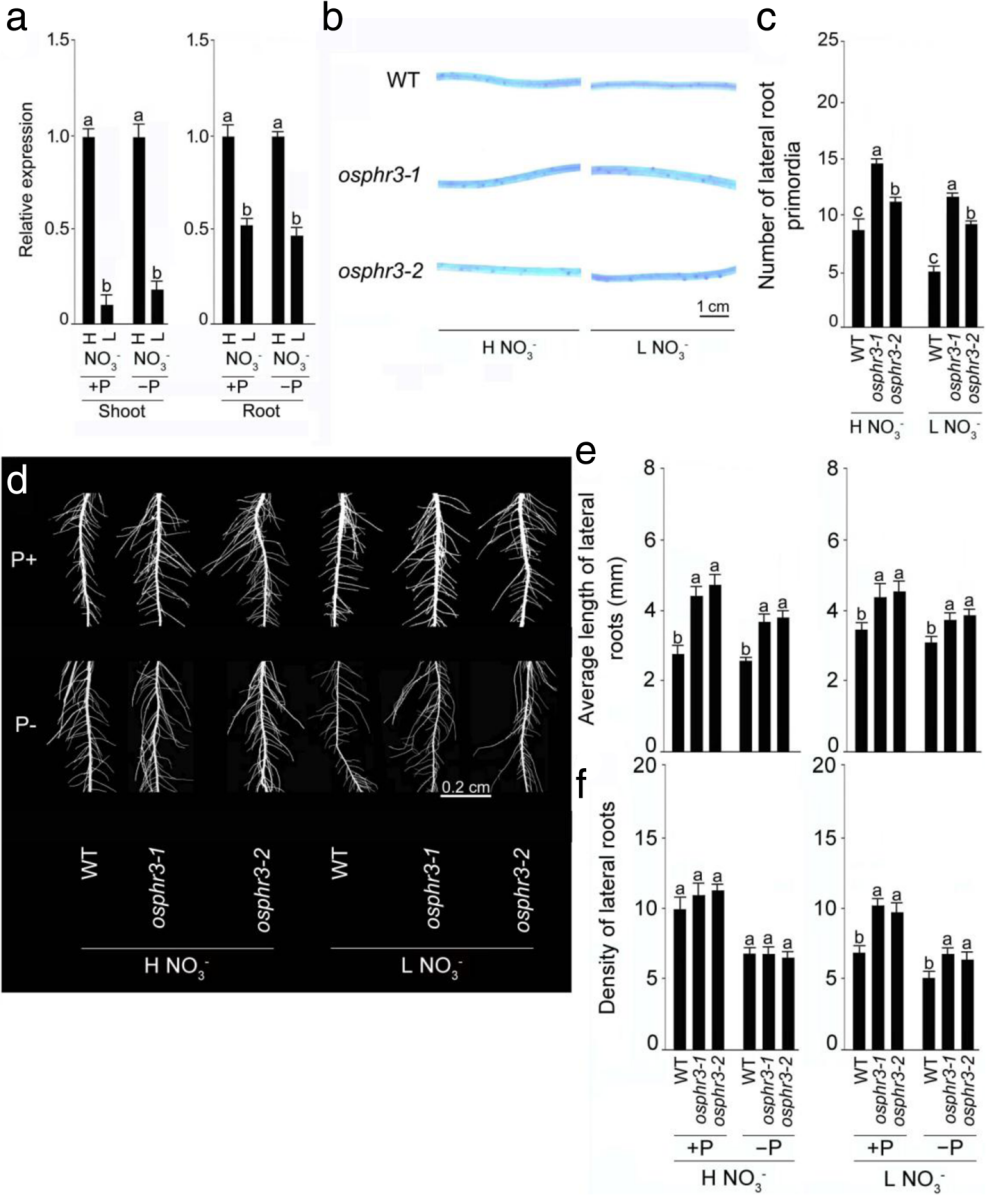
Responses of *OsPHR3* expression and lateral roots development in *osphr3* under H/L NO_3_^−^ are Pi-independent. Seeds of the WT and mutants (*osphr3–1* and *3–2*) were grown hydroponically in media comprising H NO_3_^−^ + P, H NO_3_^−^ -P, L NO_3_^−^ + P and L NO_3_^−^ -P for 10 d. **a** The relative expression level of *OsPHR3* under different NO_3_^−^ and Pi conditions. **b** Phenotype of primordia in 2–4 cm region from the tip of seminal root. **d** Seedlings showing lateral roots phenotype. Data are presented for (**c** )number of lateral root primordia, (**e**) average length and (**f**) density of lateral roots in 2–4 cm region from the tip of seminal root. Values are means ±SE (*n* = 10) and different letters on the histograms indicate that the values differ significantly (*P* < 0.05, one-way ANOVA)

Several studies have shown the prevalence of a cross-talk between sensing and signaling cascades governing homeostasis of Pi and NO_3_^−^ in Arabidopsis [38–40] and rice [41]. To know whether the relative expression of *OsPHR3* response to different NO_3_^−^ regimes depending on Pi availability, the relative expression levels of *OsPHR3* under H/L NO_3_^−^ + P and H/L NO_3_^−^ -P conditions were detected (Fig. 7a). It was found that the relative expression levels of *OsPHR*3 were reduced significantly in L NO_3_^−^ shoot and L NO_3_^−^ root compared with H NO_3_^−^ seedling under both +P and -P conditions (Fig. 7a). The wild-type and the mutants (*osphr3–1*and *osphr3–2*) were grown hydroponically under various NO_3_^−^ and NH_4_^+^ regimes for 10d (Fig. 7 and Additional file 5). To observe if there were any detectable changes in the lateral root initiation, their seminal roots (2–4 cm from the tip) were stained with methylthionine chloride (Fig. 7b, c and Additional file 5). The number of lateral root primordia was comparable between the wild-type and the mutants when grown under different NH_4_^+^ regimes (Additional file 5), while it was significantly higher in the mutants both under H NO_3_^−^ (~ 30–70%) and L NO_3_^−^ (~ 86–137%) compared with the wild-type (Fig. 7b, c). To further investigate, whether Pi availability exerts any influence on the developmental responses of the lateral roots under different NO_3_^−^ regimes, the wild-type, and mutants (*osphr3–1* and *osphr3–2*) were grown hydroponically under +P/H NO_3_^−^, -P/H NO_3_^−^, +P/L NO_3_^−^ and -P/L NO_3_^−^ conditions (Fig. 7d-f). Minor differences were observed in the lateral root phenotype of the wild-type and the mutants under all the 4 conditions tested (Fig. 7d). The average length of lateral roots of the mutants were significantly higher (~ 60–69% in +P/H NO_3_^−^, ~ 26–31% in +P/L NO_3_^−^, ~ 42–47% in +P/H NO_3_^−^ and ~ 21–25% in -P/L NO_3_^−^) in the mutants compared with their corresponding wild-type (Fig. 7e). Although the density of lateral roots of the wild-type and the mutants were comparable under +P/H NO_3_^−^ and + P/H NO_3_^−^, the values were significantly higher in the mutants compared with the wild-type under both +P/L NO_3_^−^ (~ 40–47%) and -P/L NO_3_^−^ (~ 25–33%) (Fig. 7f). These results revealed that *OsPHR3* exerts regulatory influences on the developmental responses of the lateral roots under different NO_3_^−^ regimes independent of Pi availability. However, there were no significant differences in the average length of the lateral roots of the wild-type and the mutants grown under different NH_4_^+^ regimes (Additional file 5).

### Silencing of *OsPHR3* affects N homeostasis independent of pi availability

**Fig. 8:**
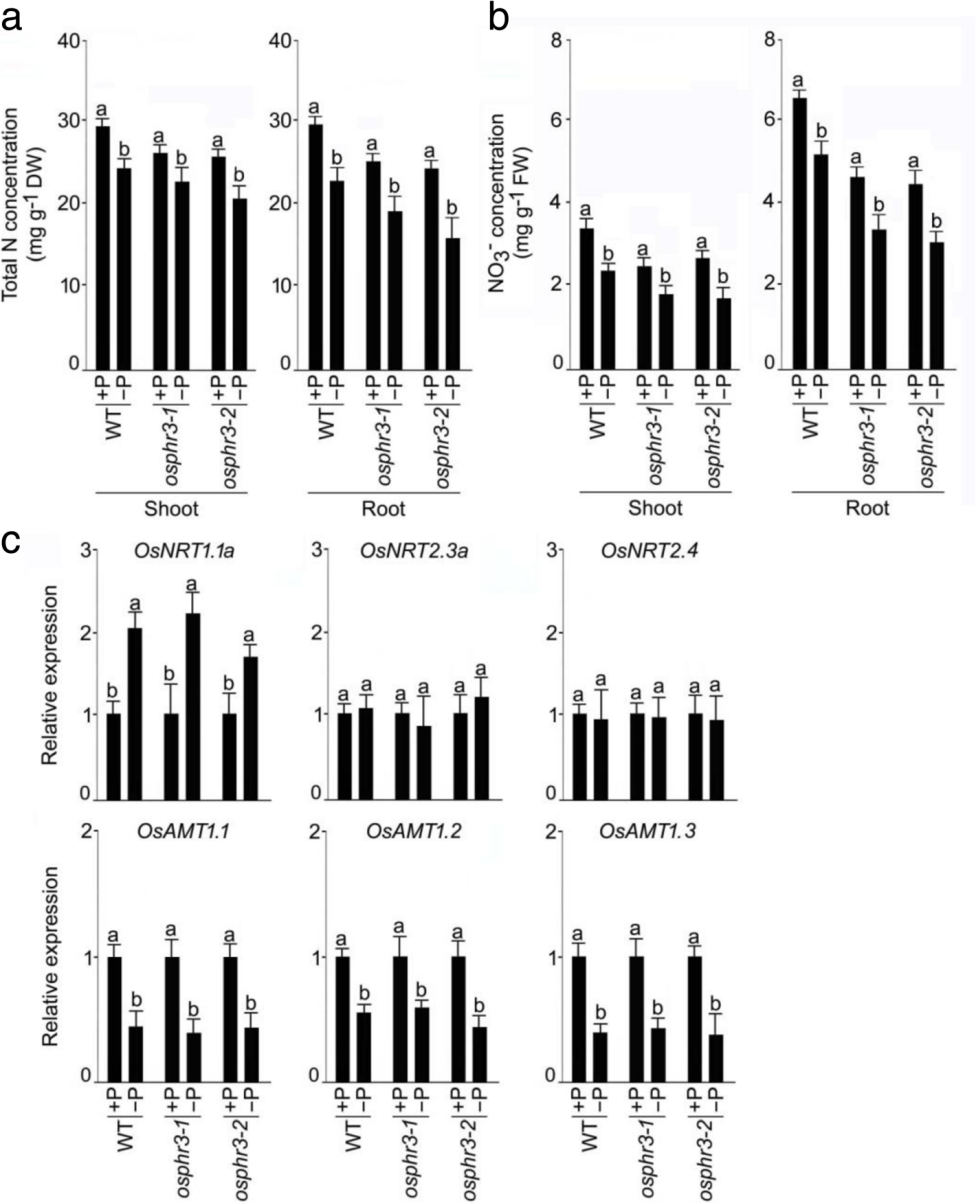
Effects of total N, NO_3_^−^ and expression of *NRTs* and *AMTs* are Pi-independent in *osphr3*. Seeds of the WT and mutants (*osphr3–1* and *3–2*) were grown hydroponically in IRRI solution for 2 weeks and then transferred to +P and -P for (**a, b**) 2 weeks and (**c**) 3 d. Shoot and root were harvested for assaying the (**a**) total N and (**b**) NO_3_^−^ concentration. **c** qRT-PCR was employed for determining the relative expression levels of genes encoding NO_3_^−^ (*OsNRT1.1a*, *OsNRT2.3a* and *OsNRT2.4*) and NH_4_^+^ (*OsAMT1.1*, *OsAMT1.2* and *OsAMT1.3*) transporters in the root. *Actin* was used as an internal control and the values for +P WT and + P mutants were normalized to 1. Values of are means ±SE (*n* = 5) and different letters on the histograms indicate that the values differ significantly (*P* < 0.05, one-way ANOVA)

Earlier studies have shown the prevalence of a cross-talk between the homeostasis of N and Pi in rice [9, 10]. The Pi and total P concentration under different N conditions were not affected by the mutation of *OsPHR3* in both shoot and root (Additional file 6)*.* T o determine the effects of Pi availability on the total N and NO_3_^−^ concentration, the wild-type and the mutants (*osphr3–1*and *osphr3–2*) were grown hydroponically under different Pi regimes for 2 weeks. Shoot and root were harvested and assayed for total N (Fig. 8a) and NO_3_^−^ (Fig. 8b) concentrations. Consistent with the earlier studies [9, 10], Pi deficiency triggered significant reductions in the total N and NO_3_^−^ concentration in the shoot and root of wild-type, *osphr3–1* and *osphr3–2* (Fig. 8a, b). Further, the mutation of *OsPHR3* reduced the total N and NO_3_^−^ concentration under both +P and –P conditions (Fig. 8a, b). The results suggested that the mutation in *OsPHR3* does not affect the regulatory mechanism governing accumulation of N under different Pi regimes. Further, the relative expression levels of the NO_3_^−^ (*OsNRT1.1a*, *OsNRT2.3a* and *OsNRT2.4*) and NH_4_^+^ (*OsAMT1.1*, *OsAMT1.2*, and *OsAMT1.3*) transporter genes were assayed in the roots of the wild-type and the mutants (*osphr3–1* and *osphr3–2*) grown hydroponically under +P and -P conditions for 3d (Fig. 8c). Pi deprivation exerted variable influences on the relative expression levels of these genes in roots of the wild-type ranging from no significant effects on *OsNRT2.3a* and *OsNRT2.4,* induction of *OsNRT1.1a* and suppression of *OsAMT1.1*, *OsAMT1.2*, and *OsAMT1.3.* It is noteworthy that the variable effects of Pi deprivation on the relative expression levels of these genes in the mutants were comparable with the wild-type (Fig. 8c). The results suggested that the mutation in *OsPHR3* affects the molecular traits governing N homeostasis independent of Pi availability.

## Discussion

In higher plants, deficiencies of Pi and/or N trigger an array of adaptive morphophysiological responses and induction or suppression of several genes belonging to different functional categories [2, 6–8]. These genes are transcriptionally regulated by a host of TFs [2, 11]. Among the TFs, the functional characterization of PHR1 in Arabidopsis [14–16, 21] and its ortholog OsPHR2 in rice [26] provided a framework of a central regulatory system governing transcriptional responses to Pi deficiency in taxonomically diverse plant species. *OsPHR3* plays an important role in improving the tolerance towards Pi deficiency [27]. *PHR1*-related *PHL1* in Arabidopsis [16] and the paralogs of *OsPHR2* i.e., *OsPHR1*, *3* and *4* in rice [27, 28] play functionally redundant roles in the maintenance of Pi homeostasis. Several studies have also shown the prevalence of a cross-talk between sensing and signaling cascades governing homeostasis of Pi and N in Arabidopsis [38–40, 42], rice [9] and maize [43]. Therefore, in this study, we investigated the likely role of *OsPHR3* in regulating the homeostasis of different forms of N under different Pi regimes.

Among different available N sources, NO_3_^−^ and NH_4_^+^ are often present in natural and cropland soils at much higher concentrations compared with other sources [44]. Therefore, effects of different forms and concentrations of N on the relative expression levels of *OsPHR3* in root and shoot was determined by employing qRT-PCR (Fig. 1). L NH_4_^+^ (in root), L NO_3_^−^ and -N (in shoot and root) triggered attenuation in the relative expression levels of *OsPHR3. OsPHR3* was significantly induced during Pi deficiency [27, 28]. Interestingly, the relative expression level of *OsPHR3* under L NH_4_^+^ was comparable with H NH_4_^+^ in the shoot (Fig. 1a). This could be due to the fact that a large amount of NH_4_^+^ assimilates locally in the root [2].

Ty1-copia retrotransposon *Tos17* is a potent tool for rice functional genomics [45]. Characterization of *Tos17* insertion mutant *osphr3* revealed the role of *OsPHR3* in exerting a regulatory influence on Pi homeostasis in rice [27]. In this study, we used *Tos17* insertion homozygous knock-out mutants *osphr3–1, osphr3–2 and osphr3–3* (Additional file 2) for deciphering the effects of the mutation in *OsPHR3* on various morphophysiological and molecular responses of rice during growth under different forms and concentrations of N. In rice, post-embryonically developed adventitious and lateral roots constitute a bulk of the root system at maturity, while embryonically developed primary and seminal roots play important roles at the seedling stage [46, 47]. Adventitious roots facilitate nutrients and water uptake and gas exchange during flooding [48]. The root biomass (+N and -N) and the average length of the adventitious roots under H NO_3_^−^, L NO_3_^−^ and L NH_4_^+^ conditions were significantly lower in these mutants compared with the wild-type (Fig. 2a-d; Additional file 3). NO_3_^−^ also acts as a signal and plays a dual role of stimulatory and inhibitory effects under mild and severe N deficiency, respectively on the total length of the lateral roots [49, 50]. Here, the number of lateral root primordia (Fig. 7b, c) and density of lateral roots (irrespective of Pi regimes) (Fig. 7f) were significantly reduced in the wild-type under L NO_3_^−^ compared with H NO_3_^−^. On the contrary, irrespective of Pi availability, average length of lateral roots was significantly higher in the wild-type under L NO_3_^−^ compared with H NO_3_^−^ (Fig. 7e). Analysis of the mutants (*osphr3–1* and *osphr3–2*) revealed negative regulatory influences of *OsPHR3* on the developmental of the number of lateral root primordia (H NO_3_^−^ and L NO_3_^−^) (Fig. 7b, c), and irrespective of Pi status, on an average length of lateral roots (H NO_3_^−^ and L NO_3_^−^) and density of lateral roots (L NO_3_^−^) (Fig. 7d-f). Auxin plays a pivotal role in the development of lateral roots [51–53] (De Smet et al., 2007, Laskowski et al., 2008, Mai et al., 2014). NRT1.1 has been shown to transport, in addition to NO_3_^−^, basipetal auxin and regulate development of the lateral root in response to the availability of external L NO_3_^−^ in Arabidopsis [54, 55]. The attenuated relative expression levels of *OsNRT1.1a* in +N and -N roots of the mutants (*osphr3–1 and osphr3–1)* compared with the wild-type (Fig. 6) suggested retarded auxin transport, which could have possibly triggered the elongation of the lateral roots in the mutants (Fig. 7e). However, the mutation in *OsPHR3* did not exhibit any influence on the lateral root development when grown under different NH_4_^+^ regimes (Additional file 5). This could be due to a more pronounced influence of NO_3_^−^ than NH_4_^+^ on the developmental responses of the lateral roots [56, 57]. The result suggested that *OsPHR3* could positively influence the acquisition of N by exerting regulatory influences on the developmental responses of ontogenetically distinct different root traits. The adverse effects of the mutation in *OsPHR3* were also evident at the grain-filling stage on the panicle development, per cent seed-set and grain yield (Fig. 2e-g). The results were in agreement with an earlier study, which reported higher grain yield in *OsPHR3* overexpression lines compared with the wild-type [27].

Significant reductions in the concentrations of total N (+N and -N shoot and root), NO_3_^−^ (+N shoot, +N and -N root) and NH_4_^+^ (-N root) in *osph3–1* and *osphr3–2* mutants compared with the wild-type suggested positive regulatory influence of *OsPHR3* on different forms and concentrations of N in a tissue-specific manner (Fig. 3a-c). It was interesting to note that the concentrations of total N and NH_4_^+^ in the shoot were comparable in the mutants and the wild-type, while all forms of N showed attenuation in -N roots (Fig. 3a-c). It is not surprising because roots are involved in sensing and acquisition of N from the soil in the form of NO_3_^−^ and NH_4_^+^ [49]. The differential effects of the mutation in *OsPHR3* were also evident in the concentration of total N in different tissues at the reproductive stage ranging from significant reduction in 6th leaf blade, increases in 1st leaf blade and seed and remained unaffected in other tissues (3rd leaf blade, culm, leaf sheath and panicle) compared with the wild-type (Additional file 4). NO_3_^−^ and NH_4_^+^ are predominant inorganic forms of N in aerated soils and anaerobic environments, respectively and their mixture is often beneficial to plants for augmenting their N content and consequently growth and development [58]. Although NH_4_^+^ is often preferred over NO_3_^−^ as the N source due to the lower energy requirement by the former for assimilation by roots [59], acquisition of NH_4_^+^ and its subsequent translocation is significantly enhanced by NO_3_^−^ availability but the former strongly suppresses influx of the latter [46, 60]. Therefore, interactions between NO_3_^−^ and NH_4_^+^ are critical for optimal utilization of N by the plants. Mutation in *OsPHR3* resulted in the attenuated influx rates of both ^15^N-NO_3_^−^ and ^15^N-NH_4_^+^ under -N condition (Fig. 4a, c). The low level of N induced the development of primary root [61]. However, the mutation of *OsPHR3* significantly reduced the primary root development and root biomass (Fig. 2). This could be one of the reasons for the lower N uptake rate in the mutants compared with the wild-type. The ratio (translocation) of ^15^NO_3_^−^ in +N root was also significantly reduced in the mutants compared with the wild-type (Fig. 4b). The results provided some explanation towards observed reductions in the concentrations of total N, NO_3_^−^ and NH_4_^+^ in -N roots of the mutants compared with the wild-type (Fig. 3a-c). On the contrary, *OsPHR3* negatively regulated mobilization of NO_3_^−^ from OLB to YLB under different NO_3_^−^ regimes (Fig. 5a) and redistribution of ^15^NO_3_^−^ (Fig. 5b). The data thus provided evidence towards the key role of *OsPHR3* in regulating the homeostasis of N, NO_3_^−^ and NH_4_^+^ under different N regimes in a tissue-specific manner.

This raised a question whether genes encoding for transporters for NO_3_^−^ (*OsNRTs*) [29–33, 62, 63] and NH_4_^+^ (*OsAMTs*) [34–36] are transcriptionally regulated by *OsPHR3*. *OsNRT1.1a* encodes a low-affinity NO_3_^−^ transporter and plays a role in the accumulation of N [63]. Whereas, *OsNRT2.4* is largely expressed in the base of the lateral root primordia, leaves, hull and in the vascular tissue of the anther and its expression is relatively much higher in the roots supplied with NO_3_^−^ compared with NH_4_^+^ solution [29]. It played a role in NO_3_^−^ regulated root growth and NO_3_^−^ distribution [62]. Transgenic rice overexpressing high-affinity NH_4_^+^ transporter *OsAMT1;1* has higher NH_4_^+^ permeability and exhibits better growth and higher yield under optimal and suboptimal NH_4_^+^ conditions [35]. *OsAMT1.3* also encodes a high-affinity NH_4_^+^ transporter, which is expressed predominantly in -N roots [36]. Here, the mutation in *OsPHR3* caused significant reductions in the relative expression levels of *OsNRT1.1a* (+N and -N conditions), *OsNRT2.4* and *OsAMT1.1* (-N condition) and but augmentation in the relative expression levels of *OsAMT1.3* (+N condition) (Fig. 6). These results suggested that the decrease of N uptake and accumulation may be due to the down-regulation of the ammonium and nitrate transporter genes in the *OsPHR3* mutants. Furthermore, NO_3_^−^ is converted to NH_4_^+^ by NO_3_^−^ reductase (NR) and nitrite reductase (NiR), and the NH_4_^+^ derived from NO_3_^−^ and/or directly acquired by the root is further assimilated into amino acids in the shoot [2]. In our study, the NO_3_^−^ reductase genes (*OsNia1* and *OsNia2*) were reduced in +N root (*OsNia1*) and –N root (*OsNia1* and *OsNia2*) in the mutants (Fig. 6). It maybe a reason which cause the strong reduction of NH_4_^+^ concentration in root of mutants under –N condition (Fig. 3c). These results of relative expression levels preliminarily explain the reduction of total N, NO_3_^−^ and NH_4_^+^ concentration, influx rate and translocation ratio in the mutants (Figs. 3 and 4). This study thus suggested a pivotal role of *OsPHR3* in regulating the expression of a subset of genes, which are involved in the maintenance of the homeostasis of NO_3_^−^, NH_4_^+^ and N. All the N treatments were carried out as described [31, 64] with slight modifications. The 2-week old wild-type and the mutants (grown hydroponically in IRRI solution) to N starvation for 3 d was to ensure the consumption of N before subjecting them to different treatments. This is a conventional practice that has been followed in our earlier studies as well [31, 64]. Among the *NRT* genes in rice, the expression of *OsNRT2.4* was significantly induced by both low N and P [65]. The *cis*-element analysis by PLACE (https://sogo.dna.affrc.go.jp/cgibin/sogo.cgi?lang=en&pj=640&action=page&page=newplace) showed that there were several Pi related *cis*-elments on the promoter of *OsNRT2.4*, such as W-box. However, there was no P1BS, which is the PHR1-specific binding sequence [14]. It suggested that *OsPHR3* may regulate the *NRT* genes in an indirect manner. The more detailed mechanism need further verification.

Earlier studies have also shown the prevalence of an antagonistic cross-talk between signaling pathways of N and Pi in rice [9] and Arabidopsis [39, 40, 42]. For instance, GARP TF HRS1 suppresses primary root growth during Pi deficiency only when NO_3_^−^ is present [40], and lower NO_3_^−^ and higher Pi concentrations promote flowering [39]. This led us to investigate whether the availability of Pi would exert any influence on the regulation of *OsPHR3* in various responses to different NO_3_^−^ or N regimes. NO_3_^−^ deficiency triggered attenuation in the relative expression of *OsPHR3* in shoot and root under both +P and -P conditions (Fig. 7a). This provided evidence towards NO_3_^−^ deficiency-mediated suppression of *OsPHR3* in the root independent of Pi availability. In terms of the development of lateral roots, it was observed that the responses of elongation and density of lateral roots in mutants to different NO_3_^−^ regimes were independent on Pi availability (Fig. 7d-f). Although Pi deficiency triggered a significant reduction in the concentration of total N and NO_3_^−^ in the shoot and root of the wild-type, the mutation in *OsPHR3* did not alter the trend (Fig. 8a, b). These results provided empirical evidences toward the regulatory influence of *OsPHR3* on the responses to NO_3_^−^ treatments and the concentration of total N and NO_3_^−^ independent of Pi status. Although the effects of Pi deficiency on the relative expression levels of these genes were differential ranging from no influence (*OsNRT2.3a* and *OsNRT2.4*), inhibitory (*OsAMT1;1*, *OsAMT1;2* and *OsAMT1;3*) and stimulatory (*OsNRT1.1a*) in the wild-type, the mutants (*osphr3–1* and *osphr3–2*) revealed a similar trend (Fig. 8b). However, the Pi and total P concentration were not affected by the mutation of *OsPHR3* under both +N and –N conditions (Additional file 6). The results were in agreement with an earlier study (Guo et al. 2015). This could possibly be due to the redundant role of *OsPHR3* with other PHR1 family members (*PHR1/2/4*) in regulating Pi homeostasis under different N regimes [27, 28]. The results provided evidence towards the regulatory influence of *OsPHR3* on these genes under different N regimes irrespective of Pi regimes.

## Conclusion

This study presented that *OsPHR3* is responsive to different forms of N irrespective of Pi regimes*.* The silencing of this gene triggered wide-spectrum effects on phenotypes during vegetative and reproductive growth phases. The analysis of total N, NO_3_^−^ and NH_4_^+^ concentrations, influx rates, translocation and distribution ratio of ^15^N, and relative expression levels of N transport and metabolism related genes suggested that silencing of *OsPHR3* regulated N homeostasis in tissue-specific manner. Further an insight into the likely roles of *OsPHR3* in regulating the lateral root development under different NO_3_^−^ regimes and N homeostasis independent on Pi availability were gained. These results from the study explain that availability of Pi did not exert any notable effects on *OsPHR3*-mediated regulatory influence on N homeostasis under different N variants and the lateral root development under different NO_3_^−^ treatments. It provide a basis for further detailed characterization of the cross-talk between N and P.

## Methods

### Plant materials and growth conditions

Wild-type rice (*Oryza sativa* L. ssp. *japonica* cv. Nipponbare) was used in the present study. The mutants *osphr3–1* (RTIM NE3007), *osphr3–2* (RTIM NE3709) and *osphr3–3* (RTIM NE3735) in Nipponbare background were obtained from the rice *Tos*17 insertion mutant database (https://tos.nias.affrc.go.jp). Homozygous mutants were identified by using a set of primers (P1-P5) in two-round semi-quantitative RT-PCR and lack of *OsPHR3* transcripts validated their fidelity (Additional files 2 and 7). Seeds of the wild-type and the mutants were grown hydroponically in IRRI solution comprising NH_4_NO_3_ (1.25 mM), CaCl_2_ (1 mM), MgSO_4_ (1 mM), Na_2_SiO_3_ (0.5 mM), K_2_SO_4_ (0.35 mM), KH_2_PO_4_ (0.3 mM), EDTA-Fe (20 μM), H_3_BO_3_ (20 μM), MnCl_2_ (9 μM), ZnSO_4_ (0.77 μM), (NH_4_)_6_Mo_7_O_24_ (0.39 μM) and CuSO_4_ (0.32 μM) with pH adjusted to 5.5. Seedlings were then transferred to nutrient solution containing different form and concentration of N: +N (2.5 mM), -N (0 mM), high NH_4_^+^ (H NH_4_^+^, 5 mM), low NH_4_^+^ (L NH_4_^+^, 0.25 mM), high NO_3_^−^ (H NO_3_^−^, 5 mM) and low NO_3_^−^ (L NO_3_^−^, 0.25 mM). These hydroponic media were maintained either under +P (Pi, 200 μM) or -P (Pi, 0 μM) condition. To inhibit nitrification, hydroponic medium containing different concentration of NH_4_^+^ was supplemented with 7 μM of dicyandiamide (C_2_H_4_N_4_). Plants were grown under controlled conditions (16 h light, 30 °C /8 h dark, 22 °C cycle and ~ 70% relative humidity).

### qRT-PCR analysis

Total RNA (~ 1 μg) was extracted from the plant tissue by using Trizol reagent (Invitrogen) and treated with RNase-free DNase (Thermoscientific). First-strand cDNA was synthesized using an oligo (dT) 18 primer and reverse transcribed using Superscript II™ Reverse Transcriptase (Invitrogen). *OsActin* (accession number AB047313) was used as an internal control and qRT-PCR analysis was performed by using SYBR Premix Ex Taq™ II (TaKaRa) in *StepOnePlus*™ Real-Time PCR *System* (Applied Biosystems). Relative expression levels of genes were computed by 2^-ΔΔ*C*^_T_ method of relative quantification [66]. The gene-specific primers used are listed in Additional file 8.

### Quantification of total N, NO_3_^−^ and NH_4_^+^

Different tissues were harvested and washed with CaSO_4_ (0.1 mM) for 1 min. Concentration of total N was determined by Kjeldahl method as described [67], while those of NO_3_^−^ and NH_4_^+^ by using a continuous-flow auto-analyzer (AutoAnalyzer 3).

### Assay for the influx and distribution of NO_3_^−^ and NH_4_^+^

Seedlings (3-d-old) of the wild-type and the mutants (*osphr3–1*and *osphr3–2*) were grown hydroponically in the IRRI nutrient solution for 2 weeks and then deprived of N for 3 d. Plants were rinsed in CaSO_4_ (0.1 mM) for 1 min and then transferred to the IRRI nutrient solution containing either 0.25 mM or 2.5 mM ^15^NO_3_^−^ (atom % ^15^N: ^15^NO_3_^−^, 60%) and 0.25 mM or 2.5 mM ^15^NH_4_^+^ (atom % ^15^N: ^15^NH_4_^+^, 60%) for 10 min and 24 h for their influx and distribution (shoot/root), respectively. In addition, to determine the redistribution of NO_3_^−^ from N-starved old to the young leaf, wild-type and the mutants were grown to the five-leaf stage. NO_3_^−^ concentration of the second leaf (old) and fourth leaf (young) of the wild-type and the mutants were analyzed. Then, the oldest leaf blade of each plant was wiped gently with a sponge and incubated in solution containing 5 mM Ca (^15^NO_3_)_2_ for 5 h. After the treatment, the youngest leaf blade (first) from the top was sampled after 24 h for determining ^15^N distribution. Plants were finally rinsed in CaSO_4_ (0.1 mM) for 1 min. Root and shoot were separated and frozen in liquid nitrogen. Tissues were ground to a fine powder, dried to a constant weight at 70 °C and ~ 10 mg dried tissue was analyzed using Isotope-ratio mass spectrometer (Thermo Fisher Scientific).

### Statistical analysis

Data were analyzed by ANOVA using SPSS 20 program (www.spss.com). Duncan’s multiple range test at *P* < 0.05 was carried out for all the experiments to determine the significance between the control and treatments.

## Additional files

Additional file 1: Comparative identity matrix and domain structure of MYB-CC family members in Arabidopsis and rice. (PDF 177 kb)

Additional file 2: Isolation and validation of *OsPHR3* mutants. (PDF 155 kb)

Additional file 3: Mutation in *OsPHR3* affects adventitious root length. (PDF 121 kb)

Additional file 4: Mutation in *OsPHR3* differentially affects total N concentration in different tissues. (PDF 129 kb)

Additional file 5: Mutation in *OsPHR3* does not affect the lateral root development under different NH_4_^+^ regimes. (PDF 154 kb)

Additional file 6: Mutation in *OsPHR3* has no effect on Pi and total P concentrations under different N regimes. (PDF 161 kb)

Additional file 7: Primers used for *osphr3* mutant identification. (DOCX 12 kb)

Additional file 8: Gene-specific primers used for qRT-PCR. (DOCX 15 kb)

## Abbreviations

MYB-CC: MYB coiled-coil
MYB-HTH: MYB helix-turn-helix
N: Nitrogen
NH_4_^+^: Ammonium
NiR: Nitrite reductase
NO_3_^−^: Nitrate
NR: Nitrate reductase
P1BS: PHR1-specific binding sequence
*phl1*: *phr1*-like1
*PHR*: *PHOSPHATE STARVATION RESPONSE 1*
Pi: Phosphate
PSR: Phosphate-starvation responsive
*PSR1*: *PHOSPHATE STARVATION RESPONSE 1*
PT: Phosphate transporter
TF: Transcription factor
