# Supplementary material for: OsPHR3 affects the traits governing nitrogen homeostasis in rice
Source: BMC Plant Biol. 2018 Oct 17;18:241. doi: 10.1186/s12870-018-1462-7 (PMC6192161; doi:10.1186/s12870-018-1462-7)
Supplement: Supplementary file 1 — Comparative identity matrix and domain structure of MYB-CC family members in Arabidopsis and rice. (PDF 177 kb) [file 12870_2018_1462_MOESM1_ESM.pdf]

A

Amino acid identity(%) (above the diagonal); Nucleotide identity(%) (below the diagonal)

	AtPHR1	AtPHL1	OsPHR1	OsPHR2	OsPHR3	OsPHR4
AtPHR1		44.51	32.74	35.61	36.71	30.03
AtPHL1	38.19		31.04	38.07	42.97	34.13
OsPHR1	37.83	38.10		43.12	33.84	31.27
OsPHR2	37.43	37.44	40.84		36.70	28.29
OsPHR3	31.25	32.94	34.55	26.06		56.96
OsPHR4	29.40	28.57	28.50	28.16	47.90	

B

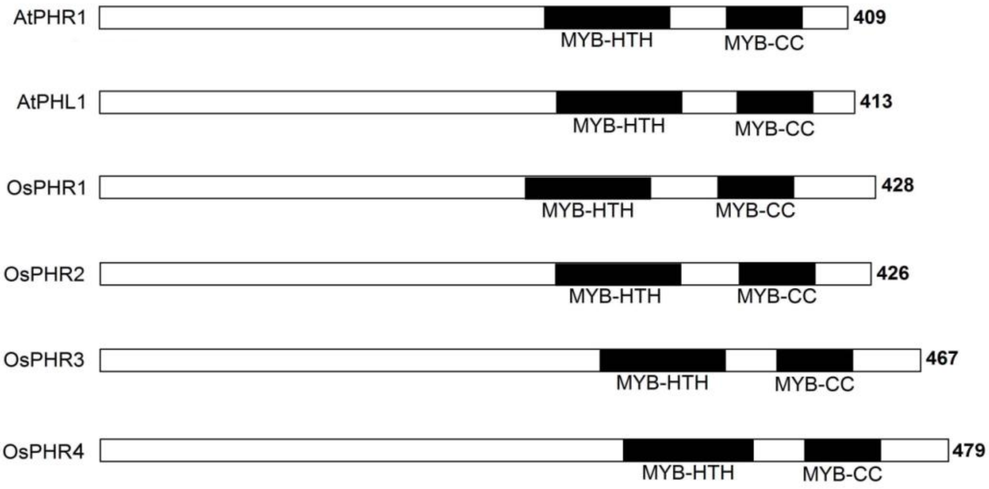

Fig. S1 Comparative identity matrix and domain structure of MYB-CC family members in Arabidopsis and rice. (A) DNAMAN 7.0 and ClusterX programs were used for the multi-sequence alignment of the nucleotides and amino acids of AtPHR1, AtPHL1 and OsPHR1-4 for determining per cent identity matrix across them. (B) MYB-HTH and MYB-CC domains in the protein are represented with black box and the number indicates total amino acids in each protein.
