# Supplementary material for: OsPHR3 affects the traits governing nitrogen homeostasis in rice
Source: BMC Plant Biol. 2018 Oct 17;18:241. doi: 10.1186/s12870-018-1462-7 (PMC6192161; doi:10.1186/s12870-018-1462-7)
Supplement: Supplementary file 2 — Isolation and validation of OsPHR3 mutants. (PDF 155 kb) [file 12870_2018_1462_MOESM2_ESM.pdf]

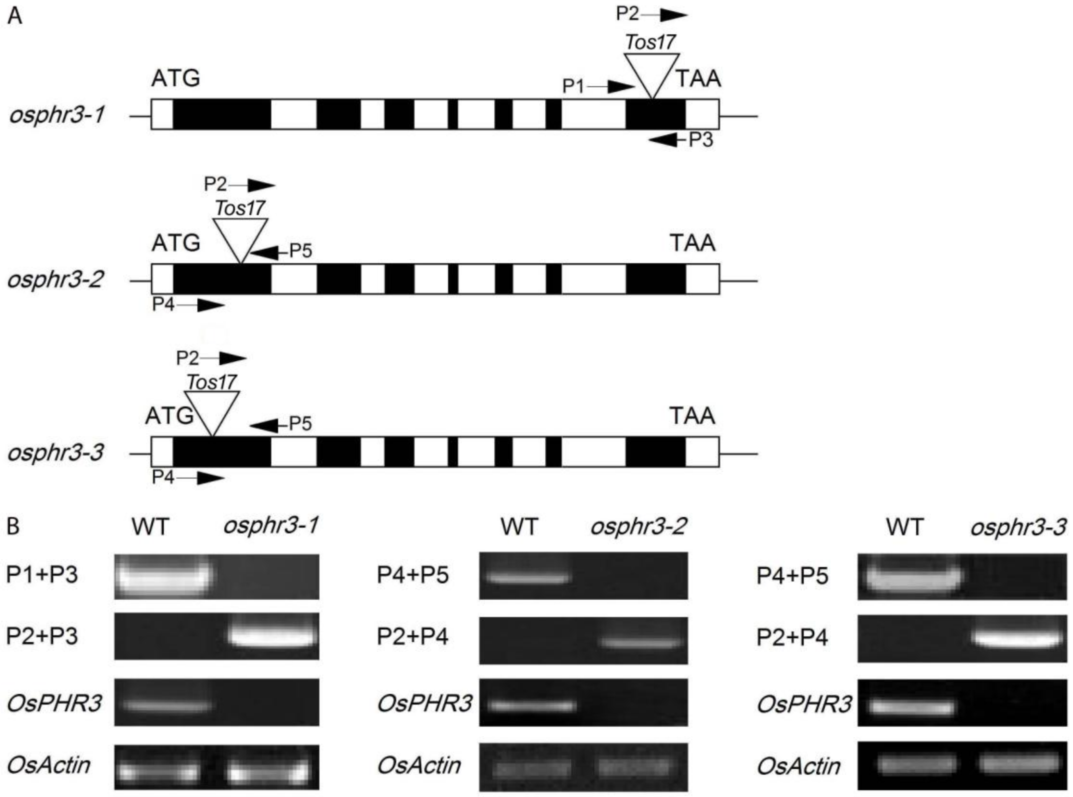

Fig. S2 Isolation and validation of *OsPHR3* mutants. (A) Schematic representation of the genomic organization of *OsPHR3* showing distribution of exons (black box) and introns (white box), Tos17 insert in the exon (white triangle) and the location of the primer sets (P1-P5) used for identifying homozygous *osphr3-1, 3-2, 3-3* knockout mutants. (B) Semi-quantitative RT-PCR analysis was employed for determining the transcript abundance of *OsPHR3* in the homozygous *osphr3-1, 3-2, 3-3* knockout mutants. *OsActin* was used as an internal control.
