# Supplementary material for: OsPHR3 affects the traits governing nitrogen homeostasis in rice
Source: BMC Plant Biol. 2018 Oct 17;18:241. doi: 10.1186/s12870-018-1462-7 (PMC6192161; doi:10.1186/s12870-018-1462-7)
Supplement: Supplementary file 3 — Mutation in OsPHR3 affects adventitious root length. (PDF 121 kb) [file 12870_2018_1462_MOESM3_ESM.pdf]

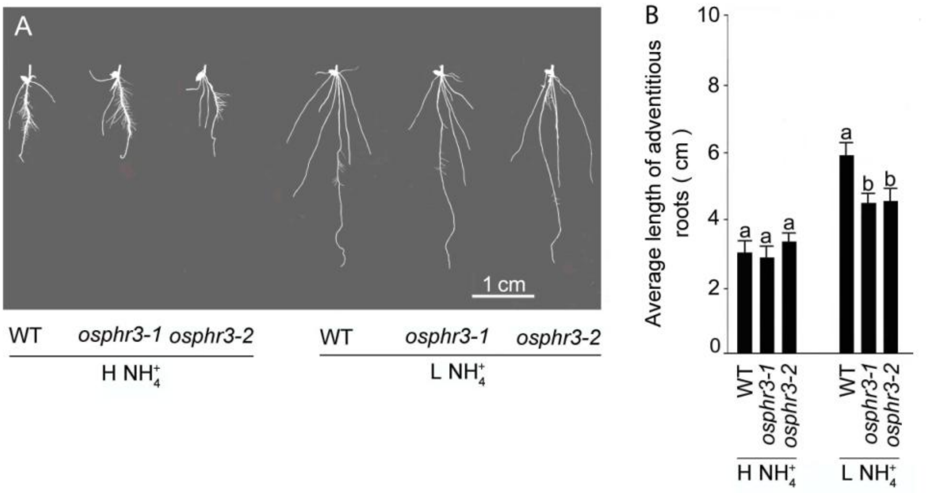

Fig. S3 Mutation in *OsPHR3* affects adventitious root length. Seedlings of the WT and the mutants (*osphr3-1* and *3-2*) were grown hydroponically in the IRRI solution supplemented with H NH_4^+ and L NH_4^+ media for 10 d. (A) Root system architecture of the WT and the mutants. (B) Data presented for an average length of adventitious roots. Values are means ±SE (n = 10) and different letters on the histograms indicate that the values differ significantly (P < 0.05, one-way ANOVA).
