# Supplementary material for: OsPHR3 affects the traits governing nitrogen homeostasis in rice
Source: BMC Plant Biol. 2018 Oct 17;18:241. doi: 10.1186/s12870-018-1462-7 (PMC6192161; doi:10.1186/s12870-018-1462-7)
Supplement: Supplementary file 4 — Mutation in OsPHR3 differentially affects total N concentration in different tissues. (PDF 129 kb) [file 12870_2018_1462_MOESM4_ESM.pdf]

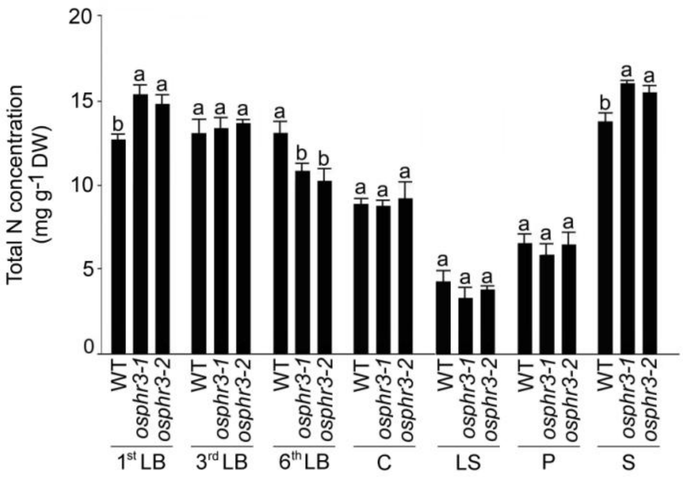

Fig. S4 Mutation in *OsPHR3* differentially affects total N concentration in different tissues. Seeds of the WT and the mutants (*osphr3-1* and *3-2*) were grown in a pot soil for 13 weeks (grain-harvest stage). Total N concentration was assayed in 1st, 3rd and 6th leaf blade (LB), culm (C), leaf sheath (LS), panicle (P) and seed (S) of the WT and the mutants (*osphr3-1* and *3-2*). The order of leaf blades is from top to bottom. Values are means ±SE (n = 5) and different letters on the histograms indicate that the values differ significantly ($P < 0.05$, one-way ANOVA).
