# Supplementary material for: OsPHR3 affects the traits governing nitrogen homeostasis in rice
Source: BMC Plant Biol. 2018 Oct 17;18:241. doi: 10.1186/s12870-018-1462-7 (PMC6192161; doi:10.1186/s12870-018-1462-7)
Supplement: Supplementary file 5 — Mutation in OsPHR3 does not affect the lateral root development under different NH4+ regimes. (PDF 154 kb) [file 12870_2018_1462_MOESM5_ESM.pdf]

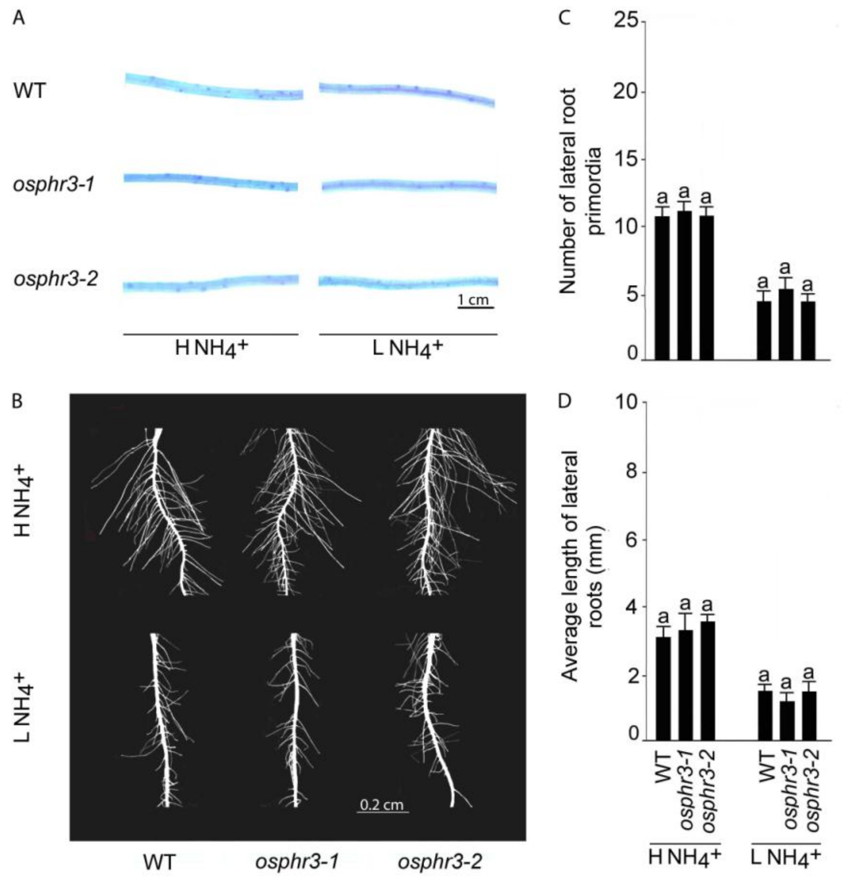

Fig. S5 Mutation in *OsPHR3* does not affect the lateral root development under different NH_4^+ regime. Seeds of the WT and the mutants (*osphr3-1* and *3-2*) were grown hydroponically in the IRRI solution supplemented with H NH_4^+ and L NH_4^+ media for 10 d. (A) phenotype of primordia in 2-4 cm region from the tip of seminal root. (B) Seedlings showing lateral root phenotype. Data are presented for (C) number of lateral root primordia in 2-4 cm region from the tip of seminal root and (D) an average length of lateral roots. Values (C and D) are means ±SE (n = 10) and different letters on the histograms indicate that the values differ significantly ($P < 0.05$, one-way ANOVA).
