# Supplementary material for: OsPHR3 affects the traits governing nitrogen homeostasis in rice
Source: BMC Plant Biol. 2018 Oct 17;18:241. doi: 10.1186/s12870-018-1462-7 (PMC6192161; doi:10.1186/s12870-018-1462-7)
Supplement: Supplementary file 6 — Mutation in OsPHR3 has no effect on Pi and total P concentrations under different N regimes. (PDF 161 kb) [file 12870_2018_1462_MOESM6_ESM.pdf]

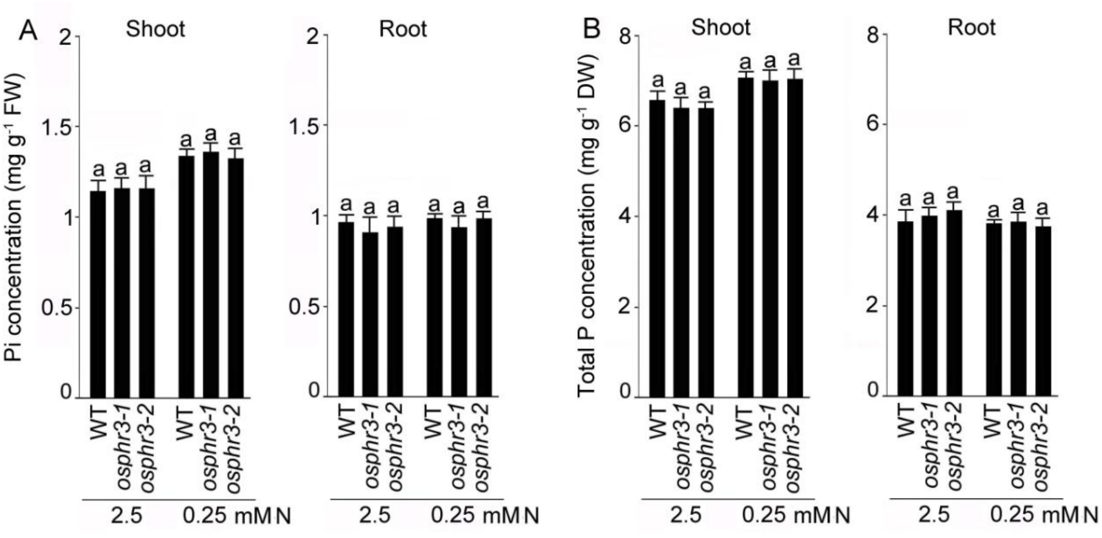

Fig. S6 Mutation in *OsPHR3* has no effect on Pi and total P concentrations under different N regimes. Seeds of the WT and mutants (*osphr3-1* and *3-2*) were grown hydroponically in IRRI solution for 2 weeks, deprived of N for 3 d and then transferred to 2.5 mM N and 0.25 mM N media for 7 d. Shoot and root were harvested. Data are presented for the concentration of (A) Pi and (B) total P concentration. Values are means ±SE (n = 4) and different letters on the histograms indicate that the values differ significantly (P < 0.05, one-way ANOVA).
