# Supplementary material for: OsPHR3 affects the traits governing nitrogen homeostasis in rice
Source: BMC Plant Biol. 2018 Oct 17;18:241. doi: 10.1186/s12870-018-1462-7 (PMC6192161; doi:10.1186/s12870-018-1462-7)
Supplement: Supplementary file 7 — Primers used for osphr3 mutant identification. (DOCX 12 kb) [file 12870_2018_1462_MOESM7_ESM.docx]

|  |  |  |  |
| --- | --- | --- | --- |
|  |  |  |  |
|  | **Primer name** | **Primer sequence** |  |
|  | P1 | GAAACCTCTTCAGCACCTT |  |
|  | P2 | AAGGGCGACCTGCGCGGCG |  |
|  | P3 | AACCAATGGCACCATCTC |  |
|  | P4 | AAAATCGCACAAGCATACACTG |  |
|  | P5 | TTCACGGCATCTACGAAACG |  |

**Table S1.** Primers used for *osphr3* mutant identification.
