# Supplementary material for: OsPHR3 affects the traits governing nitrogen homeostasis in rice
Source: BMC Plant Biol. 2018 Oct 17;18:241. doi: 10.1186/s12870-018-1462-7 (PMC6192161; doi:10.1186/s12870-018-1462-7)
Supplement: Supplementary file 8 — Gene-specific primers used for qRT-PCR. (DOCX 15 kb) [file 12870_2018_1462_MOESM8_ESM.docx]

|  |  |  |  |
| --- | --- | --- | --- |
|  |  |  |  |
|  | **Primer name** | **Primer sequence** |  |
|  | *OsPHR3*-F | CGACAGACCACCGCAACT |  |
|  | *OsPHR3*-R | CACGGCATCTACGAAACG |  |
|  | *OsNRT1.1a*-F | CAATTGGACCTATTTCGTAGCC |  |
|  | *OsNRT1.1a*-R | GCAGAAATGGTAAAACCCCA |  |
|  | *OsNRT2.3a*-F | GCCATCCACAAGATCGGTAG |  |
|  | *OsNRT2.3a*-R | TGTGGAGCTTCCCGTAGTTG |  |
|  | *OsNRT2.4-*F | CCCTTCGTCTGCAAAAGGT |  |
|  | *OsNRT2.4-*R | TACCTGGACCCGCTGAAGAA |  |
|  | *OsAMT1.1-*F | AGCGAAGGAAGAAATCACG |  |
|  | *OsAMT1.1-*R | CCAAACAGAAACTGGCAATC |  |
|  | *OsAMT1.2-*F | TTCTACGTGCTGCACAGGTTC |  |
|  | *OsAMT1.2-*R | TTGCTCCGGCGACTTTCT |  |
|  | *OsAMT1.3-*F | GTCTAGTGGAACCGGAGGAG |  |
|  | *OsAMT1.3-*R | CCTATTATACAATCACGAAACCTG |  |
|  | *OsNia1-*F | ACTGGTGCTGGTGCTTCTGG |  |
|  | *OsNia1-*R | CGGCTGGGTGTTGAGGGACT |  |
|  | *OsNia2-*F | CCAATTCTTTCATCGTGTTCT |  |
|  | *OsNia2-*R | CATGCAGCATTTCGTTTCT |  |
|  | *OsActin-*F | CAACACCCCTGCTATGTACG |  |
|  | *OsActin-*R | CATCACCAGAGTCCAACACAA |  |

**Table S2.** Gene-specific primers used for qRT-PCR.
